# Increased Intracranial Pressure during Hemodialysis in a Patient with Anoxic Brain Injury

**DOI:** 10.1155/2017/5378928

**Published:** 2017-03-20

**Authors:** Anton Lund, Mette B. Damholt, Ditte G. Strange, Jesper Kelsen, Hasse Møller-Sørensen, Kirsten Møller

**Affiliations:** ^1^Department of Neuroanaesthesiology, Rigshospitalet, University of Copenhagen, Copenhagen, Denmark; ^2^Department of Nephrology, Rigshospitalet, University of Copenhagen, Copenhagen, Denmark; ^3^Department of Neurosurgery, Rigshospitalet, University of Copenhagen, Copenhagen, Denmark; ^4^Department of Cardiothoracic Anaesthesiology, Rigshospitalet, University of Copenhagen, Copenhagen, Denmark

## Abstract

Dialysis disequilibrium syndrome (DDS) is a serious neurological complication of hemodialysis, and patients with acute brain injury are at increased risk. We report a case of DDS leading to intracranial hypertension in a patient with anoxic brain injury and discuss the subsequent dialysis strategy. A 13-year-old girl was admitted after prolonged resuscitation from cardiac arrest. Computed tomography (CT) revealed an inferior vena cava aneurysm and multiple pulmonary emboli as the likely cause. An intracranial pressure (ICP) monitor was inserted, and, on day 3, continuous renal replacement therapy (CRRT) was initiated due to acute kidney injury, during which the patient developed severe intracranial hypertension. CT of the brain showed diffuse cerebral edema. CRRT was discontinued, sedation was increased, and hypertonic saline was administered, upon which ICP normalized. Due to persistent hyperkalemia and overhydration, ultrafiltration and intermittent hemodialysis were performed separately on day 4 with a small dialyzer, low blood and dialysate flow, and high dialysate sodium content. During subsequent treatments, isolated ultrafiltration was well tolerated, whereas hemodialysis was associated with increased ICP necessitating frequent pauses or early cessation of dialysis. In patients at risk of DDS, hemodialysis should be performed with utmost care and continuous monitoring of ICP should be considered.

## 1. Introduction

Dialysis-requiring acute kidney injury (AKI) is a common occurrence in critically ill patients and is associated with increased mortality [1]. A rare but serious complication of dialysis is the so-called dialysis disequilibrium syndrome (DDS), defined as the occurrence of acute neurological symptoms attributed to cerebral edema and increased intracranial pressure (ICP) during or following dialysis [2]. Patients with acute brain injury are at increased risk of developing DDS and present a challenge when dialysis is indicated, as the need for acute dialysis must be balanced against the risk of neurological deterioration. Here, we present a case of intracranial hypertension due to DDS in a patient with anoxic brain injury and discuss the management of hemodialysis in neurocritically ill patients.

## 2. Case Presentation

A 13-year-old girl with no prior medical history was admitted to the hospital after resuscitation from cardiac arrest. The patient had collapsed during physical exercise in school, and cardiopulmonary resuscitation (CPR) was initiated immediately by school personnel. Pulseless electrical activity was the first observed prehospital rhythm. The patient was intubated by the ambulance staff, and return of spontaneous circulation was achieved after 35 minutes through CPR and adrenaline administration.

**Figure 1:**
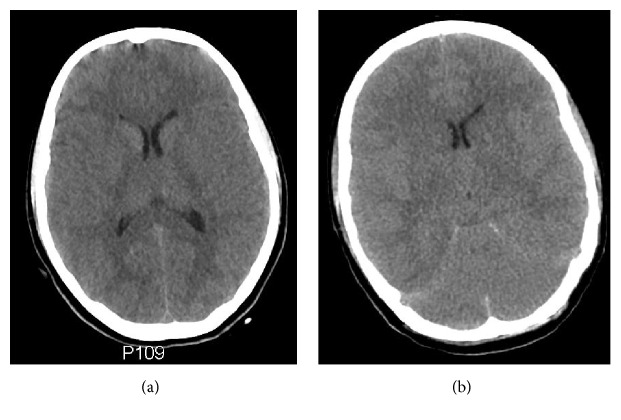
(a) Computed tomography of the brain upon arrival, showing no evidence of cerebral edema or infarction. (b) Day 3, after 7 hours of continuous renal replacement therapy: the scan now shows generalized cerebral edema with effacement of the basal cisterns and reduced grey-white matter differentiation.

Upon arrival to our emergency department, the patient was hemodynamically unstable with a mean arterial pressure (MAP) of 60–70 mmHg and heart rate of 120 bpm. Arterial blood gases showed a severe combined metabolic and respiratory acidosis with a pH of 6.7, PaCO_2_ of 83 mmHg (11 kPa), and a blood lactate of 15 mmol/L on mechanical ventilation. Bedside echocardiography revealed a dilated right ventricle and a mass in the right ventricular outflow tract suggesting a venous thrombus. Computed tomography (CT) with pulmonary angiography showed multiple peripheral emboli in both lungs, and CT of the abdomen and pelvis revealed an aneurysm of the inferior vena cava as the likely source of the emboli. CT of the brain showed no evidence of cerebral edema or infarction (Figure 1(a)). However, based on the prolonged resuscitation and decreased level of consciousness, an ICP transducer was inserted to enable detection of cerebral edema during the impending deep sedation. Initial ICP readings were normal (4 mmHg).

The patient was transferred to the cardiothoracic intensive care unit (ICU) and a cerebral perfusion pressure >60 mmHg was maintained with infusion of adrenaline and noradrenaline. Targeted temperature management aiming at 36°C for 24 hours was initiated, and the patient was started on high-dose unfractionated heparin due to the pulmonary emboli. Failed attempts to cannulate both femoral arteries resulted in bilateral hematoma formation and continued bleeding from the right femoral artery despite compression necessitated surgical exploration with repair of the artery and fasciotomy.

The day after admission, the patient had been hemodynamically stabilized and weaned off vasopressors. Sedation was gradually diminished and finally turned off. The patient demonstrated eye opening upon stimulation, pupils that were equal and reactive to light, spontaneous breathing, and a normal swallowing reflex. No spontaneous movements were observed. ICP was slightly elevated at 10–17 mmHg depending on stimulation and closely related to MAP. Laboratory analyses revealed rising levels of creatinine, urea, and potassium, and diuresis was low despite stimulation with furosemide and metolazone. Myoglobin and creatine kinase were also significantly elevated, suggesting rhabdomyolysis due to hypoxia, compartment syndrome of the right thigh, or both.

On the evening of day 3, the patient had a P-creatinine of 5.17 mg/dL (457 *μ*mol/L), P-urea of 99 mg/dL (35.4 mmol/L), and a P-potassium of 6.0 mmol/L despite infusion of glucose and insulin. The patient was visibly hypervolemic with an estimated cumulated fluid balance of +12.5 liters. ICP was stable but slightly elevated at 16–19 mmHg, and the patient was lightly sedated with remifentanil infusion. It was decided to initiate continuous renal replacement therapy (CRRT), and a double lumen dialysis catheter was placed in the right internal jugular vein. The following dialysis settings were used: Continuous venovenous hemodiafiltration, ST100 dialyzer (Gambro; surface area 1 m^2^, KUF 25 mL/(h·mmHg)), blood flow 120 mL/min, predilution flow 1000 mL/h (Prismocitrate, Gambro), dialysate flow 1000 mL/h (Prism0cal B22, Gambro), and postdilution flow 200 mL/h (Phoxillium, Gambro).

**Figure 2:**
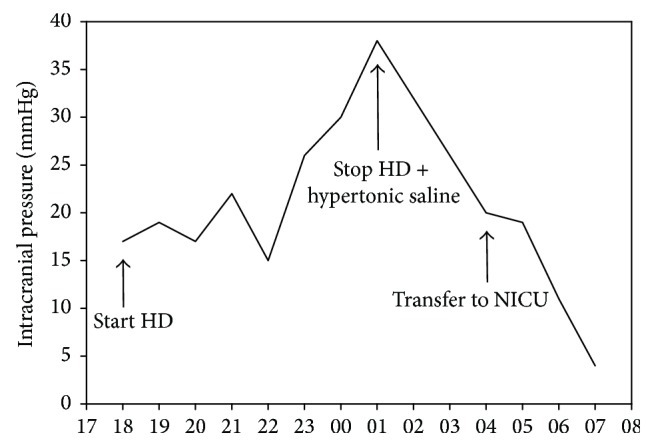
Intracranial pressure during a 13-hour period after start of continuous renal replacement therapy. *x*-axis denotes time of day. HD = hemodialysis; NICU = Neurointensive Care Unit.

Approximately seven hours after start of CRRT, ICP had increased to 38 mmHg (Figure 2); the patient had developed diverging eye axes and become unresponsive to pain (Glasgow Coma Score 3). On suspicion of cerebral edema or infarction, CRRT was stopped, 50 mL of hypertonic saline (1 mmol/mL) was administered, and the patient was sedated with propofol. CT of the brain was performed and revealed diffuse cerebral edema (Figure 1(b)). ICP decreased to 20 mmHg within a few hours. Due to the neurological deterioration and unstable ICP, the patient was transferred to the neurological ICU (NICU) and sedated with thiopentone, midazolam, and fentanyl.

On day 4, an external ventricular drain (EVD) was inserted stereotaxically based on a predicted need for further hemodialysis, upon which ICP decreased from 10 to 3 mmHg. A window of cardiovascular stability and low ICP was used as an opportunity to start careful intermittent hemodialysis. Hemodialysis was chosen over peritoneal dialysis due to hyperkalemia and the need for removal of large volumes of fluid, as well as the possibility of separating ultrafiltration and dialysis. P-creatinine was 5.92 mg/dL (523 *μ*mol/L) and P-urea 95 mg/dL (34.1 mmol/L) before start of dialysis. The patient completed 3.5 hours of isolated ultrafiltration, with ultrafiltration rate gradually increasing from 250 mL/h to 1000 mL/h and a total fluid removal of 2.5 L. Subsequently, the patient underwent 1 hour of hemodialysis without complications and with no clinically significant changes in ICP. Dialysis settings were modified to reduce the dialysis dose in an attempt to prevent DDS and were as follows: H6 dialyzer (Gambro; surface area 0.6 m^2^, KUF 33 mL/(h·mmHg)), dialysate sodium 148 mmol/L, potassium 2 mmol/L, bicarbonate 38 mmol/L, calcium 1.5 mmol/L, magnesium 0.5 mmol/L, and low dialysate flow at 300 mL/min and blood flow 150 mL/min with concurrent flows to reduce dialysis efficiency. On day 5, ultrafiltration and hemodialysis were repeated with similar settings. However, ICP gradually increased from 0 to 10 mmHg and the treatment was stopped prematurely. Similar problems were encountered on the following days, necessitating frequent pauses or early cessation of hemodialysis.

On day 8, an inferior vena cava filter was placed in order to prevent future episodes of pulmonary embolization. From day 10 and onwards, the patient was able to tolerate hemodialysis without increases in ICP. Hemodialysis was discontinued on day 21, as kidney function was rapidly returning. The patient was gradually weaned off sedation and mechanical ventilation; autonomic dysfunction ensued and was treated with baclofen and propranolol. On day 51 the patient was in a minimally conscious state (MCS) and was discharged from the NICU and transferred to a neurorehabilitation facility. Upon follow-up three months after discharge, she remained in MCS with signs of slow improvement.

## 3. Discussion

DDS occurs with a wide spectrum of clinical manifestations, ranging from mild symptoms such as headache, nausea, vomiting, blurred vision, and muscle twitching to more severe manifestations including altered mental status, coma, seizures, and death [2]. The diagnosis is largely clinical, but the syndrome has been associated with electroencephalographic abnormalities [3], and diagnostic imaging of the brain may reveal changes consistent with cerebral edema [4]. The pathophysiology of DDS remains debated, but it likely involves an osmotic shift of water across the blood-brain barrier into the brain due to a rapid removal of urea from plasma during dialysis [5]. DDS seems to be more common in children, in patients with very high blood urea concentrations, in patients with chronic kidney disease (versus AKI), and in patients undergoing their first dialysis [2, 6]. A number of cases of DDS have been reported in patients with traumatic brain injury and intracranial hemorrhage, suggesting that patients with acute brain injury are particularly susceptible to this condition. [7–13]. Several of the mentioned risk factors were present in our patient, most notably anoxic brain injury due to prolonged cardiac arrest, which resulted in cerebral edema. In the present case, ICP monitoring aided in the detection of intracranial hypertension during CRRT and led to EVD placement and a conservative dialysis strategy.

AKI is a common complication in the course of critical illness, and dialysis is often indicated due to overhydration, hyperkalemia, or severe acidosis. To correct these disturbances in a clinically appropriate time scale, hemodialysis (as opposed to peritoneal dialysis) is often the most feasible option. In patients with acute brain injury, however, caution should be exhibited due to the risk of DDS and associated intracranial hypertension. Potential protective strategies against DDS have been proposed in the literature, including slower blood and dialysate flows, smaller surface area dialyzer membranes, and high sodium and low bicarbonate concentrations in the dialysate [14]. Furthermore, continuous therapies (i.e., CRRT) have been suggested as being safer than intermittent hemodialysis in patients at risk of DDS [15, 16]. The common rationale behind these strategies is to slow down the rate of urea clearance, to reduce the change in serum osmolality, and to maintain hemodynamic stability. In our patient, however, DDS first developed during CRRT, indicating that patients are at risk even when using continuous therapies [17]. The patient was subsequently treated with intermittent hemodialysis, in which dialysis settings were modified in an attempt to reduce the risk of DDS, including separation of ultrafiltration and dialysis and modification of dialysate electrolytes. These precautions allowed sufficient fluid removal, but hemodialysis was nonetheless complicated by recurring increases in ICP, necessitating frequent pauses or early cessation of the treatment.

Hyperosmolar therapy, a mainstay in the treatment of elevated ICP, is another potential strategy for the management of DDS. The treatment can effectively reduce cerebral edema by raising plasma osmolality, thereby removing brain extracellular fluid due to creation of an osmotic gradient across the blood-brain barrier [18]. In the present case, hypertonic saline was administered during the initial ICP crisis during CRRT and was effective in reducing ICP, although discontinuation of dialysis and increased sedation were likely important as well. As a preventive measure against DDS, certain authors have suggested infusion of hypertonic saline during dialysis in high-risk patients [14]. A similar effect can be achieved using a high sodium dialysate, as employed in our case. There are, however, potential safety concerns with hyperosmolar therapy in the setting of renal failure, as insufficient renal excretion could put patients at risk of sodium overload and hypervolemia. Few studies have examined hyperosmolar therapy in this patient population, although a small retrospective study of hypertonic saline for treatment of elevated ICP in patients with renal failure found the treatment to be safe and effective [19].

## 4. Conclusion

This case highlights the inherent risks associated with hemodialysis in patients with acute brain injury. The patient in question was likely predisposed to DDS due to anoxic brain injury and cerebral edema after prolonged cardiac arrest. Increases in ICP developed both during CRRT and during careful intermittent hemodialysis, indicating that no dialysis modality confers complete protection against this complication. In high-risk patients, we suggest early start of dialysis to avoid high urea gradients during the procedure, as well as minimizing the dialysis dose. Isolated ultrafiltration seems to be well tolerated and may allow correction of fluid overload. If hemodialysis is required in the high-risk neurocritically ill patient, continuous ICP monitoring may be required to enable timely detection of and intervention against DDS.
